# Effect of Leaf Removal and Insecticide Applications on Population Densities of Leafhoppers and Mites Associated with Grapevines

**DOI:** 10.3390/insects14100791

**Published:** 2023-09-28

**Authors:** Stefan Cristian Prazaru, Giovanni dal Mas, Matteo Padoin, Denis Rizzardo, Franco Meggio, Andrea Pitacco, Alberto Pozzebon, Carlo Duso

**Affiliations:** 1 Department of Agronomy, Food, Natural Resources, Animals and Environment, University of Padua, 35020 Legnaro, Italy; giovanni.dalmas@studenti.unipd.it (G.d.M.); mpadoin98@gmail.com (M.P.); denis.rizzardo.1@studenti.unipd.it (D.R.); franco.meggio@unipd.it (F.M.); andrea.pitacco@unipd.it (A.P.); alberto.pozzebon@unipd.it (A.P.); carlo.duso@unipd.it (C.D.); 2 Centre for Research in Viticulture and Enology (CIRVE), Viale XXVIII Aprile 14, 31015 Conegliano, Italy

**Keywords:** grapevines, Cicadellidae, Phytoseiidae, IPM, cultural practices, insecticides

## Abstract

**Simple Summary:**

A study was conducted in two vineyards located in Northern Italy to investigate the effects of mechanical leaf removal, insecticide application, and their interaction on grapevine arthropods. The results showed that the use of insecticides reduced the population densities of leafhoppers and predatory mites. Mechanical leaf removal had a greater impact on the populations of predatory mites compared to leafhoppers. Interestingly, in one of the vineyards, phytophagous mite populations increased some weeks after both leaf removal and insecticide application. These effects are discussed within the IPM framework.

**Abstract:**

In this study, we tested the effects of mechanical leaf removal, insecticide application, and their interaction on leafhoppers and phytophagous and predatory mites occurring in two vineyards over three growing seasons. Leaf removal was performed in the fruit zone using a two-head pulsed air leaf remover, while insecticides were applied with a tunnel air-assisted sprayer at the maximum dose/ha recommended on the product label. Results demonstrated the efficacy of insecticide application in reducing the population densities of leafhoppers but also their detrimental effects on predatory mites. In a number of case studies, leaf removal reduced leafhopper and predatory mite densities. In one vineyard, phytophagous mite populations increased some weeks after leaf removal and insecticide application, highlighting the need to carefully consider the potential impact of vineyard management practices on non-target arthropods in the IPM framework.

## 1. Introduction

Italy has a vine-growing area of approximately 650,000 hectares [1] (Eurostat Statistics, 2022), with more than 90% dedicated to wine grape production. In 2018, Italian wine exports had a net worth of about 7.3 billion USD [2], and grapevine cultivation in all 20 regions of Italy has an impact on tourism [3]. Several factors affect grapevine cultivation, ranging from insect pest infestations to diseases associated with plant pathogenic fungi and viruses. The implementation of grapevine protection practices can have both economic and environmental consequences. In Italy and in other European countries, common grapevine pathogens such as downy and powdery mildews respectively *Plasmopara viticola* (Berk *et* Curt.) Berl. et de Toni and *Erysiphe necator* (Schwein), present significant challenges to growers. As a result, the majority of pesticide applications carried out in European vineyards are focused on the control of these diseases [4]. Insects pose a significant threat to grapevine cultivation, and, among them, grapevine moths, particularly *Lobesia botrana* (Denis & Schiffermüller) (Lepidoptera Tortricidae), are a major concern. These pests cause direct damage to grape bunches and favor the proliferation of grey mold (*Botrytis cinerea*) [4,5]. Leafhoppers (Hemiptera Cicadellidae) are also common components of grapevine fauna and can cause significant damage. *Empoasca vitis* (Göthe) is a common species that alters photosynthesis, transpiration rate, and mesophyll conductance [6], while *Zygina rhamni* Ferrari and *Erasmoneura vulnerata* (Fitch) feed on mesophyll [7,8]. The leafhopper *Scaphoideus titanus* (Ball) is the most economically important pest affecting Northern Italian vineyards and is the main vector of the phytoplasma associated with Flavescence dorée (Chuche and Thiery 2014). Mealybugs are also a major concern, as they can dramatically reduce yield and are important vectors of grapevine viruses [9,10,11,12,13]. Furthermore, spider mites (Acari: Tetranychidae) can compromise the net photosynthesis, reducing plant growth and bunch quality [14]. To achieve optimal production standards, the use of insecticides in viticulture is intense and sometimes mandatory, such as insecticides against *S. titanus* in some Italian regions [15]. However, the use of insecticides raises concerns for environmental and human health [16]. It is therefore important to develop sustainable methods to manage insect pests to ensure the health and quality of grapevine cultivation while minimizing negative impacts on the environment and human health. Cultural practices offer a promising approach to mitigating the impact of grape diseases and pests in viticulture. Research has shown that leaf canopy management practices can have a significant impact, making them a potentially effective way to reduce pesticide use [17,18,19]. Chellemi and Marois (1992) conducted a two-year experiment that demonstrated a significant reduction in the incidence of powdery mildew in plots where basal leaves were removed in the first experimental year (from 15.3% to 4.2%); this reduction doubled in the second year. Leaf removal combined with three fungicide applications achieved better control of damaged fruits compared to plots that received 11–12 fungicide applications. Similarly, English et al. (1993) found that leaf removal reduced the incidence of Botrytis bunch rot by up to 47%. Recent studies have also shown that the application of leaf removal combined with biopesticides can enhance their efficacy [19]. Overall, leaf removal has been extensively documented as having positive effects in controlling diseases and increasing grape quality in several studies [20,21,22,23,24]. These findings suggest that incorporating cultural practices into vineyard management strategies can offer a sustainable and effective way to manage grape diseases and pests while reducing the reliance on chemical pesticides. Thus, understanding the effects of these practices on both pests and beneficial insects is essential in developing sustainable pest management strategies.

### 1.1. Impact on Grapevine Arthropod Pests

The impact of leaf removal on grapevine arthropod pests has been less explored. To date, the literature has primarily focused on the effects of leaf removal on *L. botrana* populations and the consequent reduction of *B. cinerea* spread in vineyards. Vartholomaiou et al. (2008), in a two-year experiment, found that shoot thinning and/or leaf removal practices negatively impacted *L. botrana* infestation levels in vines under investigation in both years, compared to control plots [25]. Pavan et al. (2016) applied leaf removal 10 days before the start of the second-generation flight of *L. botrana* and found that larval infestation declined by about 50%. Furthermore, a positive effect was also recorded on the third generation of *L. botrana* [26]. Kiaeian et al. (2018) demonstrated that the increase in temperature due to sunlight exposure associated with bunch-zone leaf removal reduced the infestation of the European grapevine moth [27]. Tacoli et al. (2019) compared the effects of leaf removal, kaolin application, and *B. thuringensis* on the moth population. Leaf removal and kaolin and Bt application resulted in a significant decline in moth infestation levels compared to the control. Although Bt was the most effective, the efficacy of Bt and kaolin when combined with leaf removal was similar [28]. In San Joaquin Valley (CA, USA) vineyards, leaf removal proved to be an effective strategy to eliminate early-season insecticide applications against leafhoppers, with positive implications for beneficial insects, when this practice was applied to contrast bunch rot [29]. However, Tacoli et al. (2017) did not find any significant effect of leaf removal on *E. vitis* population densities when combined with kaolin spraying on different canopy zones [30].

### 1.2. Effects on Beneficial Arthropods

The effects of cultural practices on beneficials have been less investigated. Prischmann et al. (2006) reported no effects of leaf removal on predatory mite (Acari Phytoseiidae) population densities [31]. In another study, leaf removal initially decreased phytoseiid mite numbers, but their populations recovered to acceptable levels at the end of the season, even in plots where leaf removal and kaolin treatments were combined [32]. The objective of this study was to assess the impact of mechanical fruit-zone leaf removal, both alone and in combination with insecticide application, on the population densities of leafhoppers and mites occurring in two vineyards during three growing seasons.

## 2. Materials and Methods

### 2.1. Experimental Sites

This study was conducted during the 2019, 2020, and 2021 growing seasons in two conventional farms located in the province of Treviso, Veneto Region, Northeastern Italy. The farm A was in Mogliano Veneto and farm B was in the Susegana municipality. In 2019, the trials were conducted only in farm A; in 2020, in both farms; and in 2021, only in farm B. All trials were conducted on the Glera cultivar. In farm A, vines were trained with a vertical shoot position (VSP) trellis system, while vines in farm B were trained with the Sylvoz system. The trials were conducted on vineyards with a surface of about 3 hectares (farm A) and about 4 hectares (farm B), planted in 2011 and 2012, respectively.

### 2.2. Experimental Design

A factorial experimental design was applied. The factors considered were mechanical leaf removal (LR) and insecticide application (T), resulting in four treatments: 1. no leaf removal, no insecticides (CTRL-NT); 2. no leaf removal, insecticides (CTRL-T); 3. leaf removal, no insecticides (LR-NT); and 4. leaf removal, insecticides (LR-T). Tau-fluvalinate (30 mL/hL) was used in farm A and acetamiprid (150 mL/hL) in farm B to control *S. titanus* populations. Each treatment consisted of four replicates of about 50 vines. Leaf removal was done mechanically using a two-head pulsed air leaf remover (OLMI, mod. two head bilateral, Castiglione d’Asti, Italy). The forward speed was about 5 km/h with 0.8 bar operating pressure. Leaf removal was performed near the peak of the first generation of *E. vitis*, in order to minimize the total number of eggs laid and the occurrence of the second leafhopper generation. Insecticide application was always performed after leaf removal (Table 1) using a tunnel air-assisted sprayer (model “Drift Recovery”, Friuli, Agricolmeccanica, Udine, Italy).

### 2.3. Sampling

Leafhoppers and mites (both phytophagous and predatory mites) were sampled to assess the impact of leaf removal and insecticide application on grapevine arthropod communities. Before and after leaf removal and insecticide application, a total of 40 leaves per treatment (10 leaves per replicate) were randomly collected from the fruit zone approximately every 14 days, between June and August. Each leaf was immediately visually inspected for leafhoppers and all individuals were counted and removed using a brush. The leaves were then inserted into a bag and transferred to the laboratory, where grapevine arthropods were identified and quantified under a dissecting stereomicroscope (Stemi 508, Carl Zeiss Microscopy GmbH, Jena, Germany).

### 2.4. Point Quadrat Analysis (PQA)

Point Quadrat Analysis (PQA) is a commonly used method to measure vegetation patterns and structure. In this study, PQA was performed by inserting a rigid steel rod horizontally into the vineyard canopy using holes drilled every 10 cm on a 1-m wooden rod. Each treatment was repeated ten times, with four replicates carried out at random locations in the fruit zone of the canopy. During each insertion, the presence of leaves, clusters, and gaps in the canopy was noted. This information was used to calculate the leaf layer number (LLN), fraction of canopy gaps (%CG), and fraction of interior leaves (%IL), following the method described by Smart and Robinson (1991) [33]. PQA was conducted at the same time as leaf sampling, before and after leaf removal. This allowed the impact of leaf removal on the vineyard canopy to be measured and analyzed.

### 2.5. Data Analysis

To analyze the data, a repeated-measures linear mixed model was employed, using the MIXED procedure of SAS^®^ (ver. 9.4; SAS Institute Inc., Cary, NC, USA). The response variable was the number of insects or mites per leaf, with repeated measures taken at different times. Each farm’s data were analyzed separately, and sources of variation in the model included leaf removal, insecticide application, sampling time, and their interaction, which were tested using an F-test (α = 0.05). To compare the abundance of insects or mites in different treatments, multiple *t*-tests (α = 0.05) were performed on the least-square means. The Kenward–Roger method was used to estimate degrees of freedom, which can be used to calculate non-integer values for error terms. Before analysis, model assumptions were checked. The model was run on log-transformed data (n + 1), but untransformed data are shown in the figures. The SLICE option of the LSMEANS statement was used to test treatment effect variation during observation periods.

For the Point Quadrat Analysis data, the response variables were the leaf layer number (LLN), fraction of canopy gaps (%CG), and fraction of interior leaves (%IL), which were analyzed using the MIXED procedure of SAS. Treatment differences were evaluated with an F-test (α = 0.05).

## 3. Results

### 3.1. Farm A—2019

**Figure 1:**
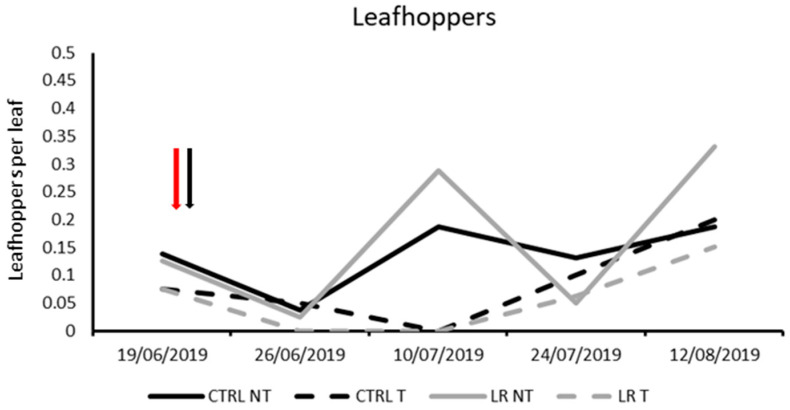
Seasonal abundance of leafhoppers in treatments under comparison in farm A during 2019 (the red arrow indicates leaf removal application, the black arrow insecticide application).

The leafhoppers found in leaf samples were represented by nymphs of *E. vitis* and *Z. rhamni*, but *E. vitis* was clearly dominant, accounting for more than 90% of the total individuals. However, the densities of leafhoppers were relatively low and thus the total number of leafhoppers was considered in the statistical analyses (Table 2). No differences were found prior to leaf removal and insecticide application (F = 0.03; d.f. = 1, 12; *p* = 0.876). In 2019, there was a decrease in leafhopper numbers with insecticide application, but the leafhopper population appeared to recover later. Leaf removal did not affect the leafhopper population (Figure 1; Table 2).

**Figure 2:**
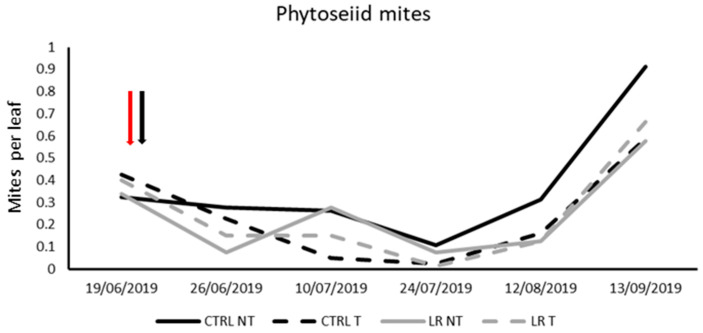
Seasonal abundance of phytoseiid mites in treatments under comparison in farm A during 2019 (the red arrow indicates the leaf removal application, the black arrow insecticide application).

Mite communities were represented mostly by predatory mites belonging to the Phytoseiidae family, particularly *Amblyseius andersoni* (Chant) and *Kampinodromus aberrans* (Oudemans). The total number of phytoseiid mites was considered in the statistical analyses (Table 3). Prior to leaf removal and insecticide application, no significant differences were found among leaf removal treatments (F = 0.06; d.f. = 1, 12; *p* = 0.817). Furthermore, neither leaf removal nor insecticide application had any effect on the number of phytoseiid mites. Only the effect of time was significant (Figure 2; Table 3).

### 3.2. Farm A—2020

**Figure 3:**
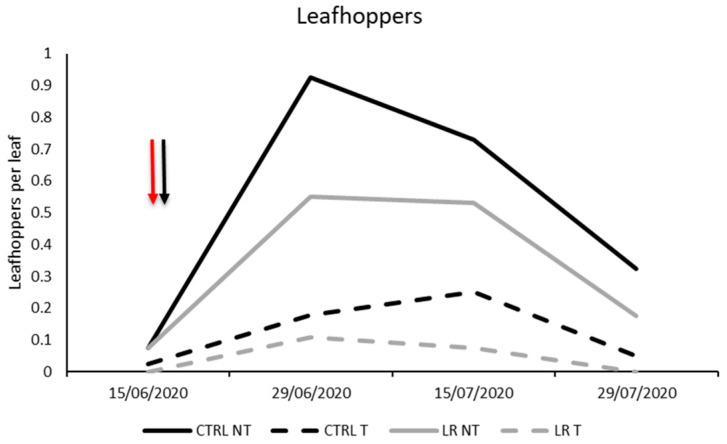
Seasonal abundance of leafhoppers in treatments under comparison in farm A during 2020 (the red arrow indicates the leaf removal application, the black arrow indicates insecticide application).

Among leafhoppers, *E. vitis* was confirmed to be the most commonly observed species, while only a few individuals of *Z. rhamni* were found. Thus, the total number of individuals was used for statistical analyses. No differences were found before leaf removal and insecticide application (F = 3.12; d.f. = 1, 12; *p* = 0.103). A population decrease was observed due to leaf removal and insecticide application, but no interactions between these factors were noticed. Additionally, the effect of time was found to be significant (Table 4; Figure 3).

**Figure 4:**
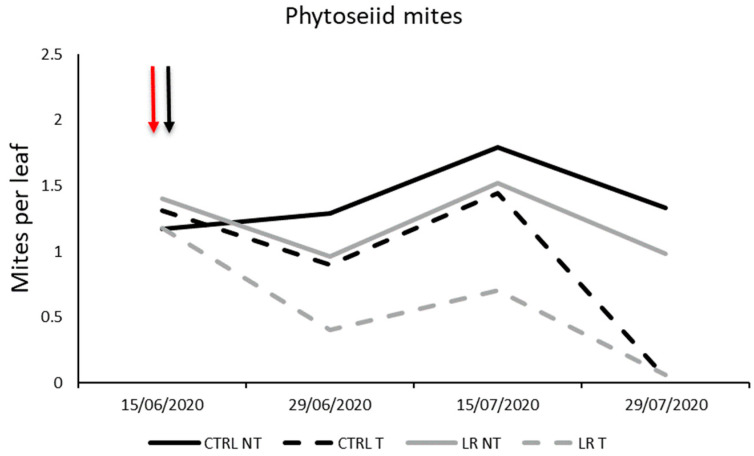
Seasonal abundance of phytoseiid mites in treatments under comparison in farm A during 2020 (the red arrow indicates the leaf removal application, the black arrow insecticide application).

*Amblyseius andersoni* and *K. aberrans* were confirmed to be the most prevalent predatory mites and their total numbers were considered in the statistical analysis. No differences among treatments were observed before leaf removal and insecticide application (F = 1.70; d.f. = 3, 12; *p* = 0.177). Then, the effects of time, leaf removal, and insecticide application were significant (Table 5) Furthermore, the interaction time × insecticide application was also significant. Both leaf removal and insecticide application resulted in a reduction in predatory mite densities (Figure 4; Table 5).

Before leaf removal in farm A, mean values for the leaf layer number (LLN) and fraction of internal leaves (IL) were similar, with values of about 6 and 68% for LLN and IL, respectively (Table 6). Leaf removal performed on June 15 caused a significant reduction in canopy density, as indicated by point quadrat parameters that differed significantly from the control (CTRL). Two weeks later, at the end of June, a significant reduction of about 2.3 leaf layers and a decrease of about 5% in the fraction of IL was observed. One month later, at the end of July, the differences in LLN and the fraction of IL between leaf removal and CTRL treatments were maintained and even increased, with a reduction of about 3.3 and 17.5% for LLN and the fraction of IL, respectively. While leaf removal was observed to significantly impact the canopy density, neither treatment showed any difference in the fraction of canopy gaps (CG), with null values observed on all sampling dates throughout the season. 

### 3.3. Farm B—2020

**Figure 5:**
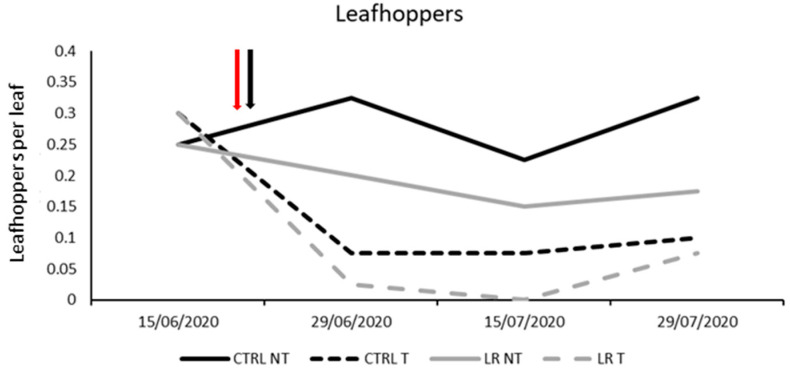
Seasonal abundance of leafhoppers in treatments under comparison in farm B during 2020 (the red arrow indicates the leaf removal application, the black arrow indicates insecticide application).

*Empoasca vitis* remained the dominant species in the vineyard, with only a few individuals of *Z. rhamni* detected. As the total densities of leafhoppers were low, their total numbers were pooled for data analysis (Table 7). No differences among treatments were found prior to leaf removal and insecticide application (F = 1; d.f. = 3, 12; *p* = 0.426). Later on, insecticide application, time, and their interaction resulted in significant differences. Insecticide application significantly reduced leafhopper densities (Figure 5, Table 7). Although leaf removal appeared to reduce leafhopper numbers, the effect was not statistically significant (*p* = 0.057).

**Figure 6:**
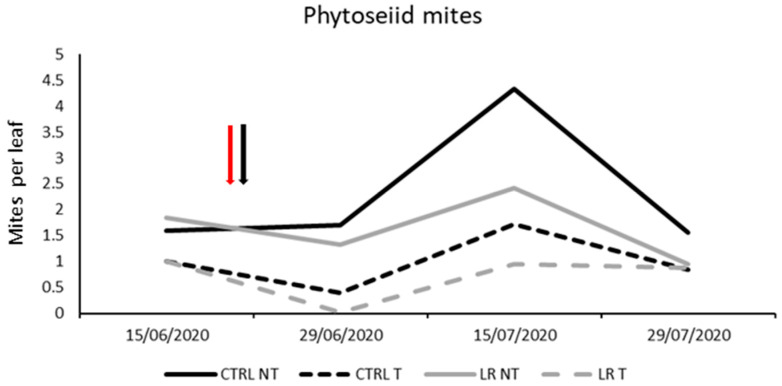
Seasonal abundance of phytoseiid mites in treatments under comparison in farm B during 2020 (the red arrow indicates the leaf removal application, the black arrow indicates insecticide application).

**Figure 7:**
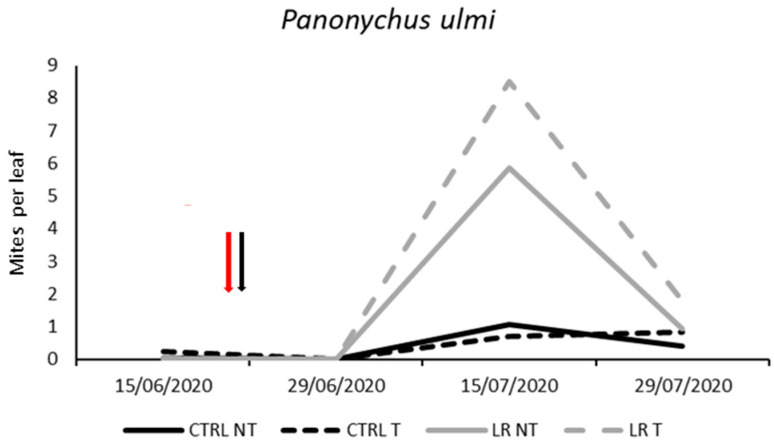
Seasonal abundance of *Panonychus ulmi* in treatments under comparison in farm B during 2020 (the red arrow indicates the leaf removal application, the black arrow indicates insecticide application).

Both predatory and phytophagous mites occurred in the vineyard (Figure 6 and Figure 7). The predatory mites detected were *A. andersoni* and *K. aberrans,* and their total numbers were included in the statistical analysis (Table 8). No significant differences were observed among treatments before leaf removal and insecticide application (F = 1.28; d.f. = 3, 12; *p* = 0.326). Later on, phytoseiid mite densities were reduced by insecticides but not by leaf-removal (*p* = 0.053); the effect of time was also significant as phytoseiid mite densities varied over time (Table 8, Figure 6). 

Phytophagous mites were represented by the spider mite *Panonychus ulmi* (Koch). No differences were found before insecticide application (F = 3.36; d.f. = 3, 12; *p* = 0.055). The effect of leaf removal and time was significant, as well as their interaction (Table 9). Higher *P. ulmi* numbers were found in the leaf removal plots compared to the control but on one date only (Figure 7). In contrast, insecticide application did not produce significant effects.

Seasonal variations in Point Quadrat Analysis-derived parameters in farm B are reported in Table 10. Similar to farm A, no differences were observed in mid-June concerning canopy density parameters prior to leaf removal, with mean values ranging between 3.3 and 3.5% and between 43.8 and 45.4% for LLN and the fraction of IL, respectively. By the end of June, after leaf removal (June 15), a reduction in canopy density was observed, with mean values of Point Quadrat Analysis-derived parameters that differed significantly among treatments. Leaf removal treatment resulted in a significant difference of about −1 and −14% in LLN and the fraction of IL, respectively, when compared to the CTRL. Two weeks later, on July 15, the CTRL canopy displayed more than doubled values of LLN and the fraction of IL compared to the leaf removal treatment, with values of +2.18 and +40% for LLN and %IL, respectively. Delayed secondary shoot development likely occurred in the fruiting zone of the canopy in leaf removal canopies against the CTRL, recovering the leaf density, with no differences in Point Quadrat Analysis-derived parameters two weeks later, during the last sampling date at the end of July. As observed for the vineyard trained as VSP in farm A, in farm B with the Sylvoz system, no differences were observed in the fraction of CG among treatments. Meanwhile, differently from the higher LLN measured in both treatments in the VSP canopies, an increasing trend in the fraction of canopy gaps was noticeable in the Sylvoz system, even if not significant.

### 3.4. Farm B—2021

**Figure 8:**
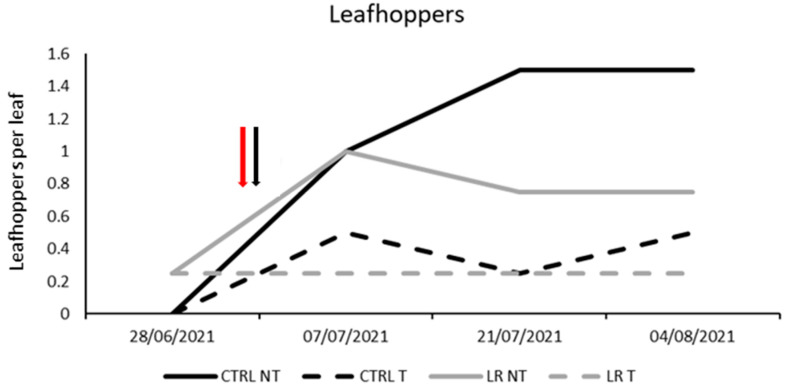
Seasonal abundance of leafhoppers in treatments under comparison in farm B during 2021 (the red arrow indicates the leaf removal application, the black arrow indicates insecticide application).

Regarding leafhoppers, no differences were found among treatments before leaf removal and insecticide application (F = 0.77; d.f. = 3, 12; *p* = 0.876). After insecticide application, a significant decrease in leafhopper densities was observed (Figure 8; Table 11).

**Figure 9:**
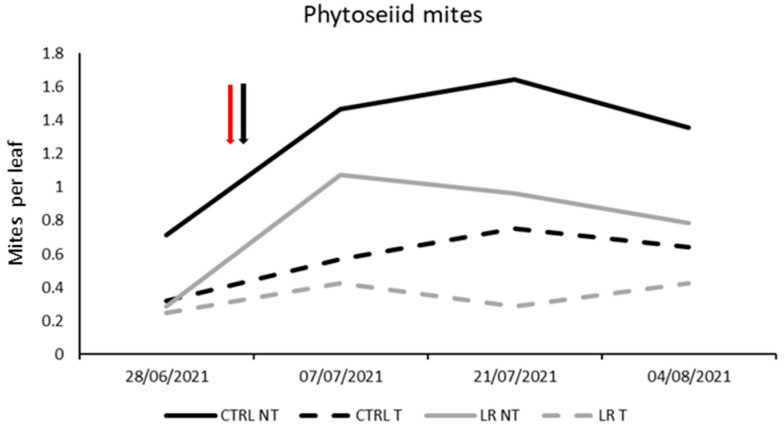
Seasonal abundance of phytoseiid mites in treatments under comparison in farm B during 2021 (the red arrow indicates the leaf removal application, the black arrow indicates insecticide application).

During the first part of the growing season, the mite communities were predominantly composed of phytoseiid mites (*A. andersoni* and *K. aberrans*). Before leaf removal and insecticide application, no differences were observed among treatments (F = 1.90; d.f. = 3, 12; *p* = 0.1425). Later, both leaf removal and insecticide application had a significant impact on phytoseiid mite densities (Figure 9; Table 12).

**Figure 10:**
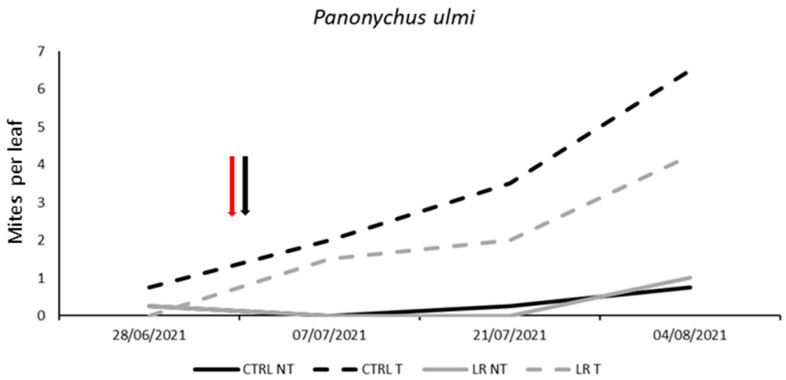
Seasonal abundance of *Panonychus ulmi* in treatments under comparison in farm B during 2021 (the red arrow indicates the leaf removal application, the black arrow indicates insecticide application).

Tetranychid populations (*P. ulmi*) increased throughout the growing season (Figure 10). No differences among treatments were found before leaf removal and insecticide application (F = 0.10; d.f. = 3, 12; *p* = 0.959). Later, the effects of insecticide application and time were significant (Figure 10; Table 13) as the tetranychid densities increased more in insecticide-treated than untreated plots (Figure 10; Table 13).

Table 14 presents the results of the canopy density measurements obtained from control and leaf removal treatments in farm B in 2021. In the previous case, similar values of Point Quadrat Analysis-derived parameters were observed in the Sylvoz system on June 28, with mean values of about 3% for LLN and 35–45% for the fraction of IL, which did not differ between treatments. However, a significant reduction in canopy density was observed on July 7 following leaf removal treatment, with mean values of about −1.5% for LLN and −22% for the fraction of IL. The leaf removal treatment maintained a sparser canopy throughout the season, while the CTRL treatment showed stable values until August, with LLN values of about 3.3–3.4% and fraction of IL values of 40–45%. However, a substantial drop was observed on July 7 and 21 in the CTRL treatment, followed by progressive leaf regrowth in the fruiting zone of the leaf removal canopy in terms of LLN and the fraction of IL. Nevertheless, the LLN and IL Point Quadrat Analysis parameters were significantly different between the leaf removal and CTRL treatments until the last sampling date, while no differences were observed for the fraction of CG among treatments on all sampling dates.

## 4. Discussion

Leafhopper densities were affected by insecticide applications in all the case studies. Insecticide formulations were based on acetamiprid and tau-fluvalinate as active ingredients, both considered effective against *E. vitis* and other leafhoppers in previous investigations [8,34,35,36,37]. Our results are consistent with those reported in other areas and suggest that the populations considered in this study did not evolve resistance to these compounds. Throughout these trials, the population density of leafhoppers consistently failed to reach the recommended action threshold for the Glera cultivar in Northeastern Italy. This threshold suggests the need for insecticide application when the population exceeds two motile forms per leaf [38,39]. We can assume that the yearly, mandatory application of at least one insecticide treatment targeting *S. titanus* [15] may have significantly influenced the leafhopper populations, making it challenging for them to attain significant density levels. The effect of leaf removal on leafhopper populations varied across the four case studies. Specifically, we observed a significant reduction in leafhopper densities in one trial where leaf removal was applied, while another trial showed a similar trend, but only approaching statistical significance (*p* = 0.057). The Point Quadrat Analysis showed that leaf removal was applied correctly, with a significant decrease in the number of interior leaves in the plots where leaf removal was applied. It is known that *E. vitis* prefers to colonize leaves located inside the canopy [40,41,42,43,44]. Furthermore, Pavan and Picotti (2009) [40] demonstrated that the number of eggs was proportional to the leaf density and thus a negative effect of leaf removal on egg laying was expected. However, in some experiments, we did not observe a significant effect of leaf removal on leafhopper populations, possibly due to low population densities or the vigorous nature of the grape cultivar used. Despite the potential benefits of leaf removal, it can have a negative impact on grape quality, limiting its usefulness in practice. To achieve a stronger effect on leafhopper populations, leaf removal may need to be repeated, but this is not always feasible or desirable [45,46].

Insecticide applications had a negative impact on predatory mite populations in three out of four case studies, with very low densities observed in the remaining case. This finding is consistent with other studies that have reported the effects of acetamiprid and tau-fluvalinate on predatory mites [47,48,49]. Acetamiprid and tau-fluvalinate are frequently used to control *S. titanus*, the main vector of phytoplasmas associated with Flavescence dorée disease, and the number of insecticide applications is increasing in Northeastern Italy. The side effects of these and other insecticides on predatory mites can favor spider mite infestations Indeed, in two out of four case studies, *P. ulmi* occurred at moderate levels. In one of these studies, spider mites reached higher densities on plots treated with insecticides than on control plots, suggesting that the use of these chemicals can disrupt the natural balance of predator–prey interactions and lead to an increase in spider mite populations.

Leaf removal was associated with a decline in predatory mite numbers in two out of four case studies. Tacoli et al. (2019) reported a similar trend, with phytoseiid mite populations initially decreasing in response to leaf removal but then recolonizing the canopy. The practice of leaf removal is known for reducing the relative humidity levels inside the canopy [50,51], which can have a negative impact on predatory mites, particularly on *A. andersoni,* which requires moderate to high relative humidity for molting and egg hatching [52]. In one case study, spider mites were found to be more abundant in leaf removal plots. It can be argued that factors inhibiting predatory mites, such as leaf removal, may favor their prey. However, it is important to note that the negative effect of leaf removal on predatory mites was, in this case, not statistically significant (*p* = 0.057).

## 5. Conclusions

Our study confirmed that insecticide applications containing acetamiprid and tau-fluvalinate were effective in controlling *E. vitis* and other leafhoppers. No resistance issues were encountered in the populations that we studied. While leaf removal had a significant impact on leafhopper populations in one of the four case studies, it did not have a substantial effect in the other three. Additionally, in two of the four case studies, leaf removal led to a decline in predatory mite numbers, likely due to decreased humidity levels. Therefore, it is important to carefully consider the potential effects of leaf removal on leafhoppers as well as on predatory and phytophagous mites and to implement sustainable and integrated pest management strategies that take into account the diverse ecological interactions that occur within the vineyard ecosystem.

## Figures and Tables

**Table 1:** Dates of leaf removal and insecticide application in the two farms and three growing seasons.

Year	2019		2020		2021	
Farm	Leaf Removal	Insecticide Application	Leaf Removal	Insecticide Application	Leaf Removal	Insecticide Application
A	19 June 2019	20 June 2019 Tau-fluvalinate (30 mL/hL)	16 June 2020	17 June 2020 Tau-fluvalinate (30 mL/hL)	\	\
B	\	\	18 June 2020	18 June 2020 Acetamiprid (150 mL/hL)	2 July 2023	2 July 2021 Tau-fluvalinate (30 mL/hL)

**Table 2:** Results of the F-test for leafhoppers occurring in farm A during 2019.

Effect	DF Num	DF Den	F-Value	*p*-Value
**Time**	**4**	**69**	**18.66**	**<0.0001**
**Insecticide**	**1**	**69**	**6.61**	**0.012**
Leaf removal	1	69	0.99	0.323
Insecticide × Leaf removal	1	69	1.77	0.188
**Time × Insecticide**	**4**	**69**	**7.10**	**<0.0001**
Time × Leaf removal	4	69	2.32	0.052
Time × Insecticide × Leaf removal	4	69	1.04	0.395

**Table 3:** Results of the F-test on phytoseiid mites occurring in farm A during 2019.

Effect	DF Num	DF Den	F-Value	*p*-Value
Time	5	72	22.34	<0.0001
Insecticide	1	72	3.35	0.071
Leaf removal	1	72	2.55	0.115
Insecticide × Leaf removal	1	72	2.96	0.089
Time × Insecticide	5	72	1.33	0.260
Time × Leaf removal	5	72	0.89	0.492
Time × Insecticide × Leaf removal	5	72	0.53	0.755

**Table 4:** Results of the F-test for leafhoppers occurring in farm A during 2020.

Effect	DF Num	DF Den	F-Value	*p*-Value
Time	3	48	27.10	<0.0001
Insecticide	1	48	76.77	<0.0001
Leaf removal	1	48	11.91	<0.0001
Insecticide × Leaf removal	1	48	1.32	0.256
Time × Insecticide	3	48	8.55	<0.001
Time × Leaf removal	3	48	1.43	0.246
Time × Insecticide × Leaf removal	3	48	0.45	0.721

**Table 5:** Results of the F-test on phytoseiid mites occurring in farm A during 2020.

Effect	DF Num	DF Den	F-Value	*p*-Value
Time	3	48	17.20	<0.0001
Insecticide	1	48	28.87	<0.0001
Leaf removal	1	48	5.09	0.029
Insecticide × Leaf removal	1	48	0.81	0.372
Time × Insecticide	3	48	2.99	0.040
Time × Leaf removal	3	48	1.04	0.385
Time × Insecticide × Leaf removal	3	48	0.65	0.587

**Table 6:** Results of the F-test for the LR effect on canopy density parameters calculated in farm A during 2020.

Site	Year	Date	Treatment	LLN	CG (%)	IL (%)
(A)	2020	15-June	CTRL	5.98	0.00	67.87
			LR	6.11	0.00	67.80
			F-value	0.047	-	0.0005
			*p*-value	0.8332	-	0.983
		29-June	CTRL	7.38	0.00	72.69
			LR	5.08	0.00	66.86
			F-value	24.28	-	10.97
			*p*-value	0.0026	-	0.016
		15-July	CTRL	5.8	0.00	67.01
			LR	4.75	0.00	58.25
			F-value	2.92	-	2.43
			*p*-value	0.1383	-	0.170
		29-July	CTRL	7.78	0.00	74.59
			LR	4.48	0.00	57.08
			F-value	27.72	-	44.30
			*p*-value	0.0019	-	0.0006

CTRL = no leaf-removal treatment; LR = leaf removal; LLN = leaf layer number; CG = fraction of canopy gaps; IL = fraction of interior leaves.

**Table 7:** Results of the F-test on leafhoppers occurring in farm B during 2020.

Effect	DF Num	DF Den	F-Value	*p*-Value
Time	3	48	4.62	0.006
Insecticide	1	48	13.71	0.001
Leaf removal	1	48	3.80	0.057
Insecticide × Leaf removal	1	48	0.61	0.439
Time × Insecticide	3	48	3.25	0.029
Time × Leaf removal	3	48	0.43	0.732
Time × Insecticide × Leaf removal	3	48	0.23	0.877

**Table 8:** Results of the F-test on phytoseiid mites occurring in farm B during 2020.

Effect	DF Num	DF Den	F-Value	*p*-Value
Time	3	48	8.68	0.0001
Insecticide	1	48	5.45	<0.0001
Leaf removal	1	48	3.93	0.053
Insecticide × Leaf removal	1	48	0.02	0.901
Time × Insecticide	3	48	2.50	0.071
Time × Leaf removal	3	48	0.95	0.422
Time × Insecticide × Leaf removal	3	48	0.52	0.668

**Table 9:** Results of the F-test for *P. ulmi* on farm B during 2020.

Effect	DF Num	DF Den	F-Value	*p*-Value
Time	3	48	30.39	<0.0001
Insecticide	1	48	2.35	0.133
Leaf removal	1	48	15.50	<0.001
Insecticide × Leaf removal	1	48	0.49	0.486
Time × Insecticide	3	48	0.50	0.683
Time × Leaf removal	3	48	10.12	<0.0001
Time × Insecticide × Leaf removal	3	48	0.65	0.589

**Table 10:** Results of the F-test for the LR effect on canopy density parameters on farm B during 2020.

Site	Year	Date	Treatment	LLN	CG %	IL %
(B)	2020	15-June	CTRL	3.35	5.00	43.81
			LR	3.53	0.00	45.38
			F-value	0.45	3	0.267
			*p*-value	0.5275	0.1340	0.624
		29-Juny	CTRL	3.00	0.00	36.01
			LR	2.06	2.78	22.05
			F-value	16.94	1.00	2.92
			*p*-value	0.0062	0.36	0.138
		15-July	CTRL	4.03	0.00	52.14
			LR	1.85	2.50	11.35
			F-value	142.81	1.00	93.95
			*p*-value	<0.0001	0.36	0.0001
		29-July	CTRL	3.38	0.00	47.42
			LR	2.50	2.50	32.97
			F-value	3.30	1.00	4.37
			*p*-value	0.1194	0.3559	0.081

CTRL = no leaf removal treatment; LR = leaf removal; LLN = leaf layer number; CG = fraction of canopy gaps; IL = fraction of interior leaves.

**Table 11:** Results of the F-test for leafhoppers on farm B during 2021.

Effect	DF Num	DF Den	F-Value	*p*-Value
Time	3	48	5.82	0.100
Insecticide	1	48	8.10	0.006
Leaf removal	1	48	0.90	0.347
Insecticide × Leaf removal	1	48	0.40	0.530
Time × Insecticide	3	48	0.97	0.416
Time × Leaf removal	3	48	0.70	0.557
Time × Insecticide × Leaf removal	3	48	0.33	0.801

**Table 12:** Results of the F-test for phytoseiid mites on farm B during 2021.

Effect	DF Num	DF Den	F-Value	*p*-Value
Time	3	48	4.13	0.011
Insecticide	1	48	23.6	<0.0001
Leaf removal	1	48	9.77	0.003
Insecticide × Leaf removal	1	48	1.54	0.220
Time × Insecticide	3	48	1.26	0.297
Time × Leaf removal	3	48	0.39	0.790
Time × Insecticide × Leaf removal	3	48	0.02	0.995

**Table 13:** Results of the F-test for *P. ulmi* on farm B during 2021.

Effect	DF Num	DF Den	F-Value	*p*-Value
Time	3	48	6.18	0.001
Insecticide	1	48	21.19	<0.0001
Leaf removal	1	48	1.63	0.207
Insecticide × Leaf removal	1	48	1.63	0.207
Time × Insecticide	3	48	3.48	0.023
Time × Leaf removal	3	48	0.14	0.935
Time × Insecticide × Leaf removal	3	48	0.21	0.891

**Table 14:** Results of the F-test for the LR effect on canopy density parameters on farm B during the 2021 season.

Site	Year	Date	Treatment	LLN	CG %	IL %
(B)	2021	28-Jun	CTRL	3.33	0.00	45.89
			LR	3.05	0.00	35.80
			F-value	1.42	-	3.66
			*p*-value	0.2779	-	0.104
		7-Jul	CTRL	3.425	0.00	45.71
			LR	1.88	2.50	23.62
			F-value	40.32	1.00	28.39
			*p*-value	0.0007	0.3559	0.002
		21-Jul	CTRL	3.35	0.00	40.35
			LR	1.55	0.00	16.07
			F-value	22.09	-	10.07
			*p*-value	0.0033	-	0.019
		4-Aug	CTRL	3.45	0.00	47.62
			LR	2.8	0.00	37.18
			F-value	9.57	-	11.49
			*p*-value	0.021	-	0.015

CTRL = no leaf removal treatment; LR = leaf removal; LLN = leaf layer number; CG = fraction of canopy gaps; IL = fraction of interior leaves.

## Data Availability

The data presented in this study are available on request from the corresponding author.
